# Association between intake of B vitamins and cognitive function in elderly Koreans with cognitive impairment

**DOI:** 10.1186/1475-2891-13-118

**Published:** 2014-12-17

**Authors:** Hyesook Kim, Ggotpin Kim, Won Jang, Seong Yoon Kim, Namsoo Chang

**Affiliations:** Department of Nutritional Science and Food Management, Ewha Womans University, Seoul, 120-750 Korea; Department of Psychiatry, Asan Medical Center, University of Ulsan, Medical College, Seoul, 138-736 Korea

**Keywords:** B vitamins, Total intake, Cognitive function, Mild cognitive impairment, Alzheimer’s disease

## Abstract

**Background:**

It is possible that blood B vitamins level and cognitive function may be affected by dietary intake of these vitamins, no study however has yet been conducted on relationships between B vitamins intake and cognitive function among elderly population in Korea. This study examined the relationship between B vitamins intake and cognitive function among elderly in South Korea.

**Methods:**

Participants consisted of 100 adults with mild cognitive impairment (MCI), 100 with Alzheimer’s disease (AD), and 121 normal subjects. Dietary intake data that included the use of dietary supplements were obtained using a 24-hour recall method by well-trained interviewers. Plasma folate and vitamin B12 concentrations were analyzed by radioimmunoassay, and homocysteine (Hcy) was assessed by a high performance liquid chromatography-fluorescence method.

**Results:**

Plasma levels of folate and vitamin B12 were positively correlated with B vitamins intake; and plasma Hcy was negatively correlated with total intake of vitamin B2, vitamin B6, vitamin B12 and folate. In the AD group, a multiple regression analysis after adjusting for covariates revealed positive relationships between vitamin B2 intake and test scores for the MMSE-KC, Boston Naming, Word Fluency, Word List Memory and Constructional Recall Tests; and between vitamin B6 intake and the MMSE-KC, Boston Naming, Word Fluency, Word List Memory, Word List Recognition, Constructional Recall and Constructional Praxis Tests. Positive associations were observed between vitamin B12 intake and the MMSE-KC, Boston Naming, Constructional Recall and Constructional Praxis Tests, and between folate intake and the Constructional Recall Test. In the MCI group, vitamin B2 intake was positively associated with the MMSE-KC and Boston Naming Test, vitamin B6 intake was positively associated with the Boston Naming Test, and folate intake was positively associated with the MMSE-KC and Word List Memory test. No associations were observed in the normal group.

**Conclusion:**

These results suggested that total B vitamins intake is associated with cognitive function in cognitively impaired AD and MCI elderly, and the association is stronger in AD patients.

## Background

A decline in cognitive function occurs as a normal part of the aging process [1]. However, a degree of cognitive impairment can be influenced by modifiable health behavior, including diet and nutrition [2]. B vitamins of folate, vitamin B2, vitamin B6 and vitamin B12 are involved in one-carbon transfer reactions such as methylation, which is necessary for the production of monoamine neurotransmitters, phospholipids and nucleotides [3] in the brain. Low levels of these B vitamins have been associated with increased homocysteine (Hcy) [4], known to have a direct neurotoxic effect [5]. Several cross-sectional [6–9] and longitudinal studies [10–13] have proposed that elevated Hcy levels may be an independent risk factor for impaired cognitive function or Alzheimer’s disease (AD). In an elderly Korean population study, it has been reported that hyperhomocysteinemia may be a significant related risk factor for mild cognitive impairment (MCI) [14].

As in Western countries [15, 16], several studies conducted in South Korea [17–19] have found relationships between the level of B vitamins in blood and cognitive function. In Korean elderly subjects, the serum folate level shows a significant positive correlation with MMSE-K [19]. The folate level in plasma and red blood cells was significantly correlated with test scores for several domains of cognitive function. Vitamin B1, vitamin B2, and vitamin B6 were positively correlated with neuropsychological function test scores, although not as strongly as folate [18].

Our previous study [20] showed that plasma folate, vitamin B12, and Hcy are associated with cognitive function in cognitively impaired (AD and MCI) elderly, and the association was stronger in patients with AD. A study in Australia investigated the relationship of serum B vitamins level and cognitive function, according to the degree of cognitive damage in AD, MCI and normal subjects [21]. However, these studies [20, 21] did not contain dietary information.

The level of B vitamins in blood, especially folate, is affected by dietary intake [22, 23]. Prospective [24] and case–control studies [25, 26] have shown that low dietary intake of B vitamins was associated with cognitive decline [24] or an increased risk of AD [25, 26]. However, null findings have been reported [27]. There is little or no data on the relationship between B vitamins intake and cognitive function among elderly population in Korea, where a folic acid fortification policy is not yet mandated.

South Korea has one of the most rapidly aging populations in the world [28]. According to a 2012 Ministry of Health and Welfare report [29], it is estimated that 9.2% of elderly aged ≥ 65 years have dementia and 27.8% have MCI. Given that the proportion of elderly with MCI and dementia in South Korea has drastically increased, more research on the correlation between B vitamins and cognitive function is needed.

This study investigated the relationship between B vitamins intake and cognitive function among normal, MCI, and AD groups in Korean elderly over 60 years of age.

## Methods

### Study participants and data collection

Study subjects aged ≥ 60 years were recruited from April 2010 to June 2011 in the Songpa district, one of 25 districts in Seoul, South Korea. All participants completed the Korean version of the Consortium to Establish a Registry for Alzheimer’s Disease (CERAD-K) assessment packet, which consists of a neuropsychological and clinical assessment battery. A total of 321 subjects, including 121 normal subjects (37.7%), 100 MCI patients (31.2%) and 100 AD patients (31.2%) were included in the final analysis. Comprehensive information is available in a previous study [20]. All subjects were interviewed by trained interviewers with caregivers present. The questionnaire was designed to obtain information on general characteristics, including age, gender, education, marital status, smoking status, and alcohol use. Disease prevalence was also investigated for vascular risk factors, including hypertension, diabetes mellitus, dyslipidemia, stroke, cardiovascular disease, and thyroid disease. Height and weight were measured, and body mass index (BMI) (kg/m^2^) was calculated as weight in kilograms divided by the square of the height in meters (kg/m^2^).

### Dietary assessment

Dietary data were collected with a 24-hour recall for intake on the day before blood sampling. Experienced, well-trained dietary interviewers instructed respondents to describe all foods and beverages consumed over the past 24 hours. The most frequently eaten food items were set as models in a defined unit and shown to subjects to increase the accuracy of reporting. Household portions in each subject’s record were converted to mass weight (in grams). Dietary intake data were analyzed using the computer-aided nutritional analysis program software (CAN-Pro version 3.0; Nutritional Assessment Program, 2006, The Korean Nutrition Society, Seoul, Korea). Evidence-based vitamin B12 values from a book of Food Values [30] and a study in South Korea [31] were added to the Can-Pro 3.0 database.

Information on brand names and consumption frequency was collected from subjects ingesting dietary supplements. Daily nutrient intake from dietary supplements was calculated using frequency and nutrient content of supplements. Dietary folate equivalent (DFE) was calculated based on 1.0 mg DFE = 1.0 mg of food folate or 0.6 mg of folic acid added to food [32]. Folate and other B vitamins intake data, including supplements, were compared with estimated average requirements (EARs) of Korean Dietary Reference Intakes (KDRI) [33].

### Statistical analysis

Data are expressed as means with standard deviation. Values for plasma folate, vitamin B12 and Hcy, and nutrient intake, were log-transformed to satisfy assumptions of normality. To examine differences in nutrient intake among the three groups, a general linear model (GLM) procedure was performed after adjusting for age, gender, BMI, marital status, education, current smoking, energy intake, thyroid disease, and vascular risk factors such as hypertension, diabetes mellitus, dyslipidemia, stroke and cardiovascular disease. Possible confounding factors were considered on the association between B vitamins intake and cognitive function; Variables 1) which were significantly different among diagnostic groups, and previously reported to have a relationship with AD (age, gender, marital status, education and current smoking), 2) which had no significant difference among diagnostic groups in the present study, nevertheless, previous studies reported to have a relationship with AD {thyroid disease and vascular risk factors (whether or not a given subject had 1 ≥ of diseases, including hypertension, diabetes mellitus, dyslipidemia, stroke and cardiovascular disease)}, and 3) which affect nutrient intake from diet {BMI, energy intake (ln)} were adjusted as covariates. Pearson’s correlation test was used to analyze correlations between nutrient intake and plasma folate, vitamin B12 and Hcy levels by group. Multiple linear regression analyses were used to examine the relationship between nutrient intake and cognitive function scores by group after adjusting for confounders. A two-tailed p-value < 0.05 was considered significant. Statistical analyses were performed using SAS software version 9.3 (SAS Institute, Cary, NC, USA).

## Results

### General characteristics and nutrient intakes

The general characteristics of participants are described in a previous study [20]. The mean age of subjects was 74.8 ± 7.2 years, and patients with AD were older than those without AD. Patients with AD were less educated, lived less often with a spouse, and comprised a higher proportion of females than those without AD. The average dietary and total nutrient intake including supplements is presented in Table 1. The total energy intake of subjects was 1490.4 ± 445.5 kcal, which was 75.1% less than the estimated energy requirement (EER) for KDRI. Mean intake of total energy, protein and folic acid was significantly lower in MCI and AD groups compared to the normal group. However, no significant difference in nutrient intake was found among all groups after adjustment for covariates of age, gender, BMI, marital status, education, current smoking, energy intake, thyroid disease, and vascular risk factors, such as hypertension, diabetes mellitus, dyslipidemia, stroke and cardiovascular disease.

**Table 1:** **Comparison of mean intake of each nutrient among AD, MCI and normal groups**

	All (*n* = 321)	AD (*n* = 100)	MCI (*n* = 100)	Normal (*n* = 121)	*p*-value	
					Unadjusted ^2^	Adjusted ^3^
Energy (kcal)						
Diet only	1487.3 ± 445.3	1450.4 ± 449.5	1447.4 ± 477.7	1550.9 ± 408.9	0.045	0.161
Total	1490.4 ± 445.5^1^	1451 ± 449.6	1450.6 ± 477.6	1555.8 ± 409.1	0.040	0.170
Protein (g/d)						
Diet only	56.0 ± 22.6	54.9 ± 23.7	53.4 ± 23.3	59.1 ± 20.9	0.022	0.460
Total	56.0 ± 22.6	54.9 ± 23.7	53.4 ± 23.3	59.1 ± 20.9	0.022	0.558
Vitamin B_2_ (mg/d)						
Diet only	0.9 ± 0 .4	0.8 ± 0.4	0.8 ± 0.4	0.9 ± 0.4	0.007	0.360
Total	2.3	1.8 ± 2.9	2.7 ± 6.2	2.4 ± 5.3	0.410	0.597
Vitamin B_6_ (mg/d)						
Diet only	1.6 ± 0.7	1.5 ± 0.7	1.5 ± 0.7	1.7 ± 0.6	0.008	0.442
Total	3.3 ± 6.6	2.4 ± 2.4	3.8 ± 7.9	3.6 ± 7.7	0.358	0.432
Vitamin B_12_ (μg/d)						
Diet only	5.7 ± 7.1	5.4 ± 6.7	5.5 ± 7.1	6.1 ± 7.4	0.170	0.586
Total	8.2 ± 14.1	6.7 ± 9.8	7.5 ± 9.9	10.0 ± 19.0	0.185	0.608
Folate (μg DFE/d)						
Diet only	437.6 ± 209.8	402.3 ± 215.5	418.7 ± 204.4	482.5 ± 203.2	0.002	0.316
Total	469.7 ± 227.1	432.9 ± 232.2	455.7 ± 220.4	511.6 ± 223.5	0.006	0.499

^1^Values are mean ± SD.

^2^Analyzed by ANOVA after log transformed.

^3^Analyzed by GLM at *p* < 0.05 after log transformed, adjusted for age, sex, BMI, marital status, education, current smoking, energy intake(ln), thyroid disease and vascular risk factors (hypertension, diabetes mellitus, dyslipidemia, stroke and cardiovascular disease) as covariates.

### Correlation between B vitamins intake and plasma concentrations of folate, vitamin B12, and Hcy

The plasma levels of folate, vitamin B12, and Hcy are reported in our previous study [20].

For total (all) subjects, after adjustment for covariates, plasma folate was positively correlated with total intake of protein (r = 0.141, p-value < 0.05), vitamin B2 (r = 0.276, p-value < 0.001), vitamin B6 (r = 0.279, p-value < 0.001) and folic acid (r = 0.339, p-value < 0.001). Plasma vitamin B12 was positively correlated with total vitamin B2 intake (r = 0.129, p-value < 0.05). Plasma Hcy was negatively correlated with total intake of vitamin B2 (r = −0.185, p-value < 0.001), vitamin B6 (r = −0.183, p-value < 0.01), vitamin B12 (r = −0.127, p-value < 0.05) and folic acid (r = −0.151, p-value < 0.01).

In the AD group, plasma folate was positively correlated with total folic acid intake only, and Hcy was negatively correlated with total vitamin B6 intake. No significant correlation was found between plasma vitamin B12 and intake of any B vitamins. In the MCI group, plasma folate was positively correlated with total intake of protein, vitamin B2, vitamin B6 and folic acid, similar to results for total subjects. Plasma vitamin B12 was positively correlated with total folic acid intake, and Hcy was negatively correlated with total vitamin B2 intake. In the normal group, plasma folate and vitamin B12 were positively correlated with total intake of protein, vitamin B2, vitamin B6 and folic acid, similar to results for total subjects. However, there was no correlation between plasma Hcy and B vitamins intake (Table 2).

**Table 2:** **Correlation coefficients between B vitamins intake and plasma homocysteine levels according to AD, MCI and normal groups**

	All (*n* = 321)	Homocysteine (μmol/L)		
		AD (*n* = 100)	MCI (*n* = 100)	Normal (*n* = 121)
Energy (kcal/d)				
Diet only	−0.040	0.126	0.166	−0.072
Total	−0.040	−0.113	0.062	−0.084
Protein (g/d)				
Diet only	−0.012	0.125	−0.063	0.010
Total	−0.065	0.125	−0.063	0.010
Vitamin B_2_ (mg/d)				
Diet only	−0.172^**^	0.069	−0.204	0.053
Total	−0.185^***^	−0.182	−0.208^*^	−0.133
Vitamin B_6_ (mg/d)				
Diet only	−0.168^**^	0.075	0.098	0.015
Total	−0.183^**^	−0.216^*^	−0.130	−0.141
Vitamin B_12_ (μg/d)				
Diet only	−0.115^*^	−0.111	−0.053	−0.019
Total	−0.127^*^	−0.173	0.069	−0.143
Folate (μg DFE/d)				
Diet only	−0.036	−0.093	0.102	−0.031
Total	−0.151^**^	−0.121	−0.104	−0.063

Data on intake and plasma level are log transformed. Adjusted for age, sex, BMI, marital status, education, current smoking, energy intake(ln), thyroid disease and vascular risk factors (hypertension, diabetes mellitus, dyslipidemia, stroke and cardiovascular disease) as covariates (*p < 0.05, **p < 0.01, ***p < 0.001).

### Association between B vitamins intake and cognitive function

In total subjects, dietary vitamin B2 intake was positively associated with the Constructional Recall Test scores, and dietary vitamin B6 intake was associated with the Boston Naming Test and Word List Memory Test scores. Folate intake was positively associated with the Word List Memory Test and Constructional Recall Test scores (Table 3).

**Table 3:** **Coefficients from multiple regression analysis between dietary B vitamins intake and neuropsychological test scores according to AD, MCI and normal groups**

Dietary Intakes		MMSE-KC	Boston naming test	Verbal fluency	Word list memory	Word list recall	Word list recognition	Constructional recall	Constructional praxis
**All** (*n* = 321)		20.6 ± 5.9	8.4 ± 3.5	9.4 ± 4.7	11.5 ± 5.5	3.1 ± 2.5	6.5 ± 3.0	3.7 ± 3.2	7.9 ± 2.7
Vitamin B_2_ (mg/d)	*β* (SE)	0.816 (0.702)	0.256 (0.451)	0.577 (0.645)	1.218 (0.670)	0.175 (0.332)	0.434 (0.428)	1.066 (0.419)	0.373 (0.341)
	*p-* value	0.246	0.571	0.371	0.070	0.599	0.312	0.012	0.275
Vitamin B_6_ (mg/d)	*β* (SE)	0.321 (0.799)	1.039 (0.510)	0.443 (0.734)	1.581 (0.760)	0.330 (0.378)	0.666 (0.486)	0.737 (0.480)	−0.059 (0.388)
	*p-*value	0.688	0.043	0.547	0.038	0.382	0.172	0.126	0.879
Vitamin B_12_ (μg/d)	*β* (SE)	0.177 (0.184)	0.023 (0.117)	0.045 (0.169)	0.293 (0.174)	0.043 (0.087)	0.082 (0.112)	0.094 (0.111)	0.121 (0.090)
	*p-*value	0.338	0.841	0.792	0.093	0.620	0.467	0.398	0.179
Folate (μg DFE/d)	*β* (SE)	0.953 (0.577)	0.157 (0.372)	0.249 (0.533)	1.095 (0.552)	0.263 (0.274)	0.395 (0.353)	0.848 (0.346)	−0.136 (0.282)
	*p-*value	0.100	0.674	0.641	0.048	0.338	0.264	0.015	0.630
**AD** (*n* = 100)									
Vitamin B_2_ (mg/d)	*β* (SE)	−0.734 (1.078)	0.185 (0.875)	0.406 (1.030)	−0.876 (1.046)	−0.015 (0.380)	−0.660 (0.897)	0.188 (0.438)	0.086 (0.717)
	*p-*value	0.498	0.833	0.694	0.405	0.969	0.464	0.669	0.905
Vitamin B_6_ (mg/d)	*β* (SE)	0.427 (1.109)	0.863 (0.894)	0.900 (1.054)	0.638 (1.077)	0.415 (0.387)	0.292 (0.924)	0.472 (0.447)	0.597 (0.733)
	*p-*value	0.701	0.337	0.395	0.555	0.287	0.753	0.294	0.418
Vitamin B_12_ (μg/d)	*β* (SE)	0.481 (0.268)	0.269 (0.216)	0.285 (0.261)	0.375 (0.261)	0.063 (0.087)	0.086 (0.226)	0.162 (0.107)	0.325 (0.180)
	*p-*value	0.077	0.216	0.278	0.154	0.472	0.705	0.135	0.075
Folate (μg DFE/d)	*β* (SE)	−0.457 (0.829)	−0.689 (0.669)	0.168 (0.792)	−0.368 (0.807)	0.237 (0.291)	−0.547 (0.689)	0.394 (0.334)	−0.814 (0.544)
	*p-*value	0.583	0.306	0.832	0.649	0.417	0.430	0.242	0.138
**MCI** (*n* = 100)									
Vitamin B_2_ (mg/d)	*β* (SE)	1.705 (1.089)	0.517 (0.762)	1.082 (1.027)	2.293 (1.145)	0.704 (0.575)	1.461 (0.682)	1.380 (0.684)	1.133 (0.587)
	*p-*value	0.121	0.499	0.295	0.048	0.224	0.035	0.047	0.057
Vitamin B_6_ (mg/d)	*β* (SE)	−0.006 (1.251)	0.583 (0.863)	−0.779 (1.168)	0.781 (1.323)	0.172 (0.657)	1.083 (0.784)	1.205 (0.782)	−0.454 (0.677)
	*p-*value	0.996	0.502	0.506	0.557	0.794	0.171	0.127	0.504
Vitamin B_12_ (μg/d)	*β* (SE)	−0.310 (0.261)	−0.236 (0.187)	0.139 (0.252)	0.078 (0.269)	−0.055 (0.139)	0.168 (0.168)	−0.026 (0.172)	−0.094 (0.148)
	*p-*value	0.238	0.209	0.582	0.772	0.691	0.319	0.879	0.529
Folate (μg DFE/d)	*β* (SE)	1.817 (0.933)	0.193 (0.659)	0.868 (0.887)	2.137 (0.984)	0.662 (0.495)	1.388 (0.586)	1.022 (0.594)	0.092 (0.517)
	*p-*value	0.055	0.770	0.330	0.033	0.185	0.020	0.089	0.859
**Normal** (*n* = 121)									
Vitamin B_2_ (mg/d)	*β* (SE)	0.020 (0.904)	−0.730 (0.699)	−0.551 (1.133)	1.215 (1.077)	−0.436 (0.573)	0.339 (0.554)	0.532 (0.807)	−0.100 (0.517)
	*p-*value^2)^	0.983	0.298	0.628	0.262	0.448	0.541	0.511	0.847
Vitamin B_6_ (mg/d)	*β* (SE)	−0.460 (1.032)	1.434 (0.791)	0.544 (1.295)	2.484 (1.215)	0.047 (0.656)	0.627 (0.631)	−0.789 (0.921)	−0.474 (0.589)
	*p-*value	0.657	0.073	0.675	0.043	0.943	0.323	0.394	0.423
Vitamin B_12_ (μg/d)	*β* (SE)	0.139 (0.259)	−0.083 (0.194)	−0.620 (0.316)	0.212 (0.309)	0.063 (0.163)	−0.146 (0.156)	0.038 (0.230)	0.213 (0.145)
	*p-*value	0.593	0.670	0.052	0.495	0.700	0.351	0.870	0.146
Folate (μg DFE/d)	*β* (SE)	0.480 (0.741)	0.074 (0.576)	−1.373 (0.922)	0.630 (0.887)	−0.550 (0.468)	−0.230 (0.455)	0.067 (0.664)	0.262 (0.424)
	*p-*value	0.518	0.898	0.139	0.479	0.242	0.615	0.920	0.538

Data on intake and plasma level are log transformed. Adjusted for age, gender, BMI, marital status, education, current smoking, energy intake (ln), thyroid disease and vascular risk factors (hypertension, diabetes mellitus, dyslipidemia, stroke and cardiovascular disease).

In AD subjects, no association was observed between cognitive function scores and any dietary parameters. In MCI subjects, dietary vitamin B2 intake was positively associated with the Word List Memory, Word List Recognition and Constructional Recall Test scores; and dietary folate intake was positively associated with the Word List Memory and Word List Recognition Test scores. In normal subjects, dietary vitamin B6 intake was positively associated with the Word List Memory Test scores.

The association between total B vitamins intake and cognitive function scores in each group is presented in Table 4. In total subjects, total vitamin B2 intake was positively associated with the MMSE-KC and Boston Naming Test scores, and total vitamin B6 intake was associated with the Boston Naming Test scores. Folic acid intake was positively associated with the MMSE-KC, Word List Memory Test and Constructional Recall Test scores. In AD subjects, total vitamin B2 intake was positively associated with the MMSE-KC, Boston Naming Test, Word Fluency, Word List Memory Test and Constructional Recall Test scores; and total vitamin B6 intake was positively associated with the MMSE-KC, Boston Naming Test, Word Fluency, Word List Memory, Word List Recognition Test, Constructional Recall Test and Constructional Praxis Test scores. Total vitamin B12 intake was positively associated with the MMSE-KC, Boston Naming Test, Constructional Recall Test and Constructional Praxis Test scores; and total folic acid intake was positively associated with the Constructional Recall Test scores. In MCI subjects, total vitamin B2 intake was positively associated with the MMSE-KC and Boston Naming Test scores; and total vitamin B6 intake was positively associated with the Boston Naming Test scores. Total folic acid intake was positively associated with the MMSE-KC and Word List Memory Test scores. No association was observed between cognitive function scores and any dietary parameters in the normal group.

**Table 4:** **Coefficients from multiple regression analysis between total B vitamins intake and neuropsychological test scores according to AD, MCI and normal groups**

Total Intakes		MMSE-KC	Boston naming test	Verbal fluency	Word list memory	Word list recall	Word list recognition	Constructional recall	Constructional praxis
**All** (*n* = 321)		20.6 ± 5.9	8.4 ± 3.5	9.4 ± 4.7	11.5 ± 5.5	3.1 ± 2.5	6.5 ± 3.0	3.7 ± 3.2	7.9 ± 2.7
Vitamin B_2_ (mg/d)	*β* (SE)	0.581 (0.265)	0.376 (0.170)	0.182 (0.245)	0.284 (0.255)	−0.055 (0.126)	0.192 (0.162)	0.079 (0.161)	0.190 (0.129)
	*p-* value	0.029	0.028	0.458	0.266	0.664	0.237	0.625	0.143
Vitamin B_6_ (mg/d)	*β* (SE)	0.609 (0.323)	0.647 (0.205)	0.317 (0.298)	0.395 (0.310)	−0.067 (0.153)	0.294 (0.197)	0.008 (0.196)	0.202 (0.157)
	*p-*value	0.060	0.002	0.288	0.204	0.664	0.137	0.969	0.201
Vitamin B_12_ (μg/d)	*β* (SE)	0.288 (0.173)	0.160 (0.111)	0.131 (0.159)	0.306 (0.164)	0.002 (0.082)	0.073 (0.106)	0.089 (0.105)	0.163 (0.085)
	*p-*value	0.098	0.150	0.413	0.063	0.979	0.490	0.397	0.055
Folate (μg DFE/d)	*β* (SE)	1.358 (0.553)	0.573 (0.357)	0.574 (0.512)	1.165 (0.530)	0.211 (0.264)	0.412 (0.339)	0.768 (0.333)	0.047 (0.271)
	*p-*value	0.015	0.110	0.263	0.029	0.425	0.226	0.022	0.863
**AD** (*n* = 100)									
Vitamin B_2_ (mg/d)	*β* (SE)	1.040 (0.426)	0.783 (0.347)	0.835 (0.411)	0.882 (0.418)	0.089 (0.154)	0.683 (0.360)	0.436 (0.172)	0.391 (0.289)
	*p-*value	0.017	0.027	0.045	0.038	0.566	0.061	0.013	0.180
Vitamin B_6_ (mg/d)	*β* (SE)	1.476 (0.526)	1.213 (0.425)	1.393 (0.502)	1.521 (0.508)	0.251 (0.191)	1.113 (0.441)	0.652 (0.211)	0.734 (0.356)
	*p-*value	0.006	0.005	0.007	0.004	0.191	0.014	0.003	0.042
Vitamin B_12_ (μg/d)	*β* (SE)	0.656 (0.247)	0.448 (0.200)	0.417 (0.244)	0.487 (0.245)	0.003 (0.092)	0.138 (0.215)	0.252 (0.099)	0.425 (0.167)
	*p-*value	0.010	0.028	0.090	0.050	0.978	0.524	0.012	0.013
Folate (μg DFE/d)	*β* (SE)	0.619 (0.765)	0.059 (0.622)	0.946 (0.725)	0.378 (0.745)	0.203 (0.269)	0.129 (0.639)	0.683 (0.303)	−0.226 (0.509)
	*p-*value	0.420	0.925	0.196	0.614	0.452	0.840	0.027	0.657
**MCI** (*n* = 100)									
Vitamin B_2_ (mg/d)	*β* (SE)	0.666 (0.400)	0.561 (0.275)	0.699 (0.373)	0.519 (0.428)	0.106 (0.213)	0.159 (0.257)	0.043 (0.258)	0.214 (0.220)
	*p-*value	0.010	0.045	0.065	0.229	0.620	0.537	0.870	0.334
Vitamin B_6_ (mg/d)	*β* (SE)	0.761 (0.497)	1.008 (0.331)	0.569 (0.467)	0.332 (0.532)	−0.014 (0.264)	0.258 (0.318)	−0.067 (0.319)	0.080 (0.273)
	*p-*value	0.129	0.003	0.227	0.534	0.959	0.420	0.835	0.769
Vitamin B_12_ (μg/d)	*β* (SE)	−0.187 (0.265)	−0.035 (0.191)	0.398 (0.252)	0.191 (0.272)	−0.080 (0.140)	0.106 (0.171)	−0.107 (0.173)	−0.119 (0.150)
	*p-*value	0.482	0.855	0.117	0.485	0.569	0.535	0.538	0.430
Folate (μg DFE/d)	*β* (SE)	2.155 (0.920)	0.951 (0.648)	1.330 (0.876)	2.231 (0.977)	0.577 (0.494)	0.936 (0.592)	0.780 (0.595)	−0.040 (0.515)
	*p-*value	0.021	0.146	0.132	0.025	0.246	0.118	0.193	0.938
**Normal** (*n* = 121)									
Vitamin B_2_ (mg/d)	*β* (SE)	0.358 (0.320)	−0.076 (0.250)	−0.436 (0.401)	−0.195 (0.385)	−0.147 (0.204)	−0.073 (0.197)	−0.158 (0.287)	0.225 (0.183)
	*p-*value^2)^	0.266	0.761	0.279	0.613	0.472	0.710	0.584	0.220
Vitamin B_6_ (mg/d)	*β* (SE)	0.151 (0.373)	0.057 (0.290)	−0.230 (0.467)	−0.040 (0.446)	−0.121 (0.236)	−0.047 (0.228)	−0.366 (0.332)	0.180 (0.213)
	*p-*value	0.686	0.843	0.623	0.929	0.609	0.836	0.272	0.400
Vitamin B_12_ (μg/d)	*β* (SE)	0.115 (0.224)	−0.093 (0.168)	−0.273 (0.270)	−0.020 (0.268)	−0.008 (0.141)	−0.154 (0.134)	−0.027 (0.199)	0.246 (0.125)
	*p-*value	0.609	0.579	0.054	0.942	0.957	0.254	0.892	0.051
Folate (μg DFE/d)	*β* (SE)	0.455 (0.585)	0.042 (0.561)	−1.425 (0.895)	0.348 (0.864)	−0.358 (0.457)	−0.313 (0.441)	0.057 (0.646)	0.470 (0.410)
	*p-*value	0.438	0.941	0.114	0.688	0.435	0.479	0.930	0.255

Data on intake are log transformed. Adjusted for age, gender, BMI, marital status, education, current smoking, energy intake (ln), thyroid disease and vascular risk factors (hypertension, diabetes mellitus, dyslipidemia, stroke and cardiovascular disease).

When we additionally adjusted for intakes of antioxidant nutrient such as vitamin C, vitamin E and β-carotene, the positive association between B vitamins intake and cognitive function still existed. A stronger association was shown in patients with AD compared to normal and MCI subjects. This suggests that the positive association between B vitamins intake and cognitive function in our study was not likely due to an increased B vitamins intake in these elderly whose antioxidant nutrient consumption was high compared with their counterparts (data not shown).

## Discussion

Dietary and total B vitamins intake was negatively correlated with plasma Hcy levels and positively associated with cognitive function scores, with different associations according to cognitive status. Positive associations between total B vitamins intake and cognitive function were observed for vitamin B2, vitamin B6, vitamin B12 and folic acid in the AD group, and for vitamin B2, vitamin B6 and folate in the MCI group. No association was observed between vitamin B intake and cognitive function in the normal group. Results are similar to our previous finding [20] that plasma folate, vitamin B12, and Hcy are associated with cognitive function in cognitively impaired (AD and MCI) elderly. A stronger association was shown in patients with AD compared to normal and MCI subjects and cannot be explained. Results indicated that plasma folate and vitamin B12 were associated with Hcy and cognitive function as reported by previous studies [7, 9, 15, 20], and dietary intake of Hcy-lowering vitamins of vitamin B2, vitamin B6, vitamin B12 and folate was also related.

A negative correlation between plasma Hcy and B vitamins intake among elderly is supported by previous reports [34, 35]. As vitamin B2, vitamin B6, vitamin B12 and folate are metabolically interrelated in one-carbon metabolism, it is not surprising that the intake of vitamin B2, vitamin B6, vitamin B12 and folate is inversely related to plasma Hcy. Hcy is a byproduct of a sulfur-containing amino acid methionine, which is formed from the universal methyl donor SAM during methylation of biomolecules and hydrolysis of S-adenosylhomocysteine to Hcy. The resulting Hcy is remethylated to methionine by methylenetetrahydrofolate, generated in the one-carbon metabolism cycle where Hcy-lowering B vitamins are metabolically interrelated [36].

Plasma Hcy level was differed significantly among diagnostic groups; however, no differences in dietary intake of vitamin B2, vitamin B6, vitamin B12 and folate were observed among three groups. A U.K. study reported that the mean intake of folate, vitamin B6, and B12 did not differ significantly between normal and AD patients, but plasma Hcy levels were influenced by intake of these vitamins [37]. The Baltimore Longitudinal Study of Aging reported that folate intake at or above the recommended dietary allowance (RDA) is associated with a reduced risk of AD, but no association was found to vitamin B6 and B12 intake [26]. Other prospective studies found no relationship between the intake of other B vitamins and AD risk [27, 38].

Cognitive function test scores were positively associated with intake of vitamin B2, vitamin B6, vitamin B12 and folate in the AD group; vitamin B2, vitamin B6 and folate in the MCI group; and none in the normal group.

To the best of our knowledge, no study had previously investigated an association between B vitamins intake and cognitive function in AD patients. Intervention studies reported that B vitamins supplementation {Folic acid (5 mg/day) and B12 (1 mg/day)} could improve cognitive performance, but only in mild to moderately demented AD patients with elevated Hcy levels at the baseline [39, 40]. Aisen et al. [41] reported that in mild AD treatment with high-dose B vitamins supplements {Folic acid (5 mg/day), B6 (25 mg/day) and B12 (1 mg/day)} did slow cognitive decline, whereas in moderate AD it did not.

In the MCI group, intake of vitamin B2, vitamin B6 and folate was positively associated with cognitive function test scores. There have been no reports on the relationship between B vitamins intake and cognitive function in MCI patients. However, a study in Korea investigating associations with serum vitamin levels and cognitive function found a relationship between serum folate level and MMSE-K scores [19]. An intervention study on MCI subjects reported that B vitamins supplementation {folic acid (0.8 mg/day), B12 (0.5 mg/day), B6 (20 mg/day)} stabilized the executive function and improved global cognition, episodic and semantic memory, and global clinical dementia rating scores in patients with elevated baseline Hcy levels [42]. In contrast, another study found that supplementation did not improve cognitive function [43]. These negative results could be explained that the subjects were not stratified for baseline homocysteine.

In the normal group, no association was observed between B vitamins intake and cognitive function scores. This is in line with conflicting results of previous studies. A UK study [27] reported no association between B vitamins intake and cognitive function scores in normal elderly patients. In Korea, two studies reported a positive relationship between vitamin B2 intake and cognitive function scores including MMSE-K in normal Korean elderly subjects [44, 45]. Studies demonstrated that supplementation with folic acid, B12, and B6 improved cognitive function such as immediate and delayed memory, information processing speed, and sensorimotor speed [46–48]; and vitamin B12 supplementation in elderly with cobalamin deficiency resulted in reduced motor function scores [49].

Results of this study showed that vitamin B6 intake was associated with the greatest number of cognitive function indicators. Daily intake of vitamin B6 was 1.6 ± 0.7 mg, and 33% of subjects consumed less than EAR for KDRI (data not shown). Seo et al. [50] reported that the average daily intake of vitamin B6 in Korean elderly residing in rural areas was 1.1 ± 0.7 mg (80.1% of RNI, Recommended Nutrient Intake), lower than our study. Chang et al. [51] reported that more than 70% of elderly people in rural Korea had inadequate functional vitamin B6 status, as measured by erythrocyte aspartate transaminase activity coefficients. Therefore, significant measures to improve nutritional status in folate, vitamin B12, and vitamin B6 among Korean elderly must be considered, including the promotion of consuming a diet rich in folate and B vitamins.

This study had potential limitations, in addition to those presented in our previous study. First, our study was observational in nature and cannot prove causality. Although we attempted to control for the effects of the major identified predictors of cognitive function, B vitamins consumption could be a marker for unrecognised factors that affect cognitive function. Second, results may be difficult to interpret and apply to the general population, as a random sample was not utilized. Third, holotranscobalamine, a better indicator of vitamin B12 status, was not measured; and serum vitamin B2 and B6 levels were not measured, only data on dietary intake. Despite limitations, and to the best of our knowledge, this is the first study investigating the relationship between cognitive function scores and dietary intake by cognitive status.

## Conclusion

An association between B vitamins intake and cognitive function was stronger in cognitively impaired AD and MCI patients than in normal elderly in Korea. Further prospective studies are warranted to investigate causal relationships of cognitive function with nutritional status, including B vitamins intake.
